# Exploring Nurses’, Preschool Teachers’ and Parents’ Perspectives on Information Sharing Using SDQ in a Swedish Setting – A Qualitative Study Using Grounded Theory

**DOI:** 10.1371/journal.pone.0168388

**Published:** 2017-01-11

**Authors:** Elisabet Fält, Anna Sarkadi, Helena Fabian

**Affiliations:** Department of Women’s and Children’s Health, Uppsala University, Uppsala, Sweden; TNO, NETHERLANDS

## Abstract

Evidence-based methods to identify behavioural problems among children are not regularly used within the Swedish Child healthcare. A new procedure was therefore introduced to assess children through parent- and preschool teacher reports using the Strengths and Difficulties Questionnaire (SDQ). This study aims to explore nurses’, preschool teachers’ and parents’ perspectives of this new information sharing model. Using the grounded theory methodology, semi-structured interviews with nurses (n = 10) at child health clinics, preschool teachers (n = 13) and parents (n = 11) of 3-, 4- and 5-year-old children were collected and analysed between March 2014 and June 2014. The analysis was conducted using constant comparative method. The participants were sampled purposively within a larger trial in Sweden. Results indicate that all stakeholders shared a desire to have a complete picture of the child's health. The perceptions that explain why the stakeholders were in favour of the new procedure—the ‘causal conditions’ in a grounded theory model—included: (1) Nurses thought that visits after 18-months were unsatisfactory, (2) Preschool teachers wanted to identify children with difficulties and (3) Parents viewed preschool teachers as being qualified to assess children. However, all stakeholders had doubts as to whether there was a reliable way to assess children’s behaviour. Although nurses found the SDQ to be useful for their clinical evaluation, they noticed that not all parents chose to participate. Both teachers and parents acknowledged benefits of information sharing. However, the former had concerns about parental reactions to their assessments and the latter about how personal information was handled. The theoretical model developed describes that the causal conditions and current context of child healthcare in many respects endorse the introduction of information sharing. However, successful implementation requires considerable work to address barriers: the tension between normative thinking versus helping children with developmental problems for preschool teachers and dealing with privacy issues and inequity in participation for parents.

## Introduction

### Background

The Swedish Child Healthcare Services (CHS) reach more than 95 per cent of the 0–5-year-old population [1]. One of the objectives of the CHS is to detect developmental and mental health problems in children and provide interventions accordingly [2]. Yet, in an overview of Swedish research, the Royal Swedish Academy of Sciences pointed out the lack of data on the mental health of young children [3]. Therefore, research that address challenges in measuring preschool aged children’s mental health was identified as a field of special importance. Behavioural problems that present early may become persistent and increase the risk for later negative academic [4,5], social and mental health outcomes [6] and therefore need to be identified and addressed. Early identification of developmental and behavioural problems can improve the child’s quality of life as well as result in socio-economic benefits [7–9]. In fact, economists claim that investments to improve children’s health and development in early childhood are the most compelling investment a society can make since the cost is returned many times over, from a lifetime perspective [10].

All Swedish parents with children aged 6 and under are offered CHS free of charge. The CHS in Sweden offers a regular programme including health and developmental check ups at Child Health Clinics (CHC) by public health nurses and general practitioners. These routine visits occur frequently during the child’s first 18 months, but become yearly visits once the child turns 3, from which time parents are invited for a check up in connection with the child’s birthday. Apart from the nurse’s observations during the brief visit, the assessment is also based on parents’ own description of how the child is functioning at home and in preschool. In Sweden, eight out of ten 1–5-year-old children attend preschool [11], and the Swedish preschool is of high quality and has well-educated staff. Thus, although it is the CHSs task to monitor children's health, behaviour and development, it is the preschools that possess much of the information needed to make a comprehensive assessment. This is also emphasised by the Swedish National Board of Health and Welfare, suggesting that health monitoring of preschool children requires increased communication between healthcare and preschool systems [12].

Implementation of a structured assessment tool to assess children’s mental health within the CHS and in preschool could serve many purposes. Such method could provide opportunity to increase knowledge of the mental health of Swedish 3- to 5-year-olds and also possibility for preschool teachers to provide nurses with important information in order to strengthen the CHSs assessment of the child. A multi-informant approach including preschool teacher assessments could reduce the risk of mental health or behaviour problems that remain unidentified. Current methods within the CHS rely solely on parents’ statements regarding the children’s everyday functioning, in order to recognise children in need of further evaluation. Available research suggests that mental health symptoms could be left undetected if only parental information is considered [13]. However, implementation of a structured assessment tool in preschool is complex since preschool teachers do not work with structured forms regularly and have rejected such methods previously, fearing that children can be stigmatised [14,15]. Instead, teachers invite parents to annual developmental talks about the child’s everyday life, leaving the parents to decide whether to raise any issues with the CHC-nurse.

### The information sharing process under study

As part of a population-based cluster-randomised trial focusing on preventing behavioural problems in children [16], a method of information sharing between preschools and the CHS was introduced. The method, a strategy for achieving increased communication between healthcare and preschools, by sharing knowledge about the individual child, was performed using the Strengths and Difficulties Questionnaire (SDQ) [17], which was completed by parents and preschool teachers. Since preschool teachers expressed concerns regarding the procedure, detailed discussions between the researchers and the preschool leadership were held before initiation of the trial, in order to come to an agreement on a convenient procedure for the information sharing process. The preschool leadership advocated a routine where parents would personally give the SDQ forms to their child’s preschool teachers, thus, ensuring the preschool teachers that it was them who wanted an assessment of their child.

The CHC nurse sent study information and three SDQs to the parents about three weeks prior to the child’s annual health and developmental check up at 3-, 4- and 5-years. Participating parents or guardians (both when applicable) were asked to complete the questionnaire and to take the third questionnaire to the child´s preschool for the teachers to complete. The teachers then sent the completed SDQ to the nurse at the CHC, and the parents returned their completed SDQs when attending the check up. The questionnaires were then reviewed by the nurse during the check up. The SDQ scores were not calculated at any stage of the process. However, a coloured score sheet indicating items with high scores within the different subscales was provided for the nurses’ convenience to identify possible areas of concern. The completed SDQs served only as basis for the nurses’ assessment and as a discussion document at the CHC visit. The nurses did not discuss the SDQ assessment with the teachers unless there were issues that needed to be clarified. Thus, information sharing using SDQ provided nurses with a quick and easy method to access the preschool teachers’ valuable knowledge about the child. The process also gives parents a possibility to take part in the preschool SDQ answers as well as an opportunity to discuss the parent and preschool SDQs with the nurse before referrals or any other actions are agreed upon. Moreover, this process might initiate and facilitate verbal communication between CHC nurses and preschool teachers when there are actual concerns regarding a child. Consequently, the information sharing process is an effort towards increased communication between nurses, teachers and parents.

Internationally, SDQ is commonly used and is a valid tool for identification of mental health problems in preventive healthcare [18]. Nevertheless, previous research has indicated that introduction of the SDQ in educational settings can be problematic due to preschool teachers’ negative attitudes towards structured assessments of children [14,15]. The SDQ was still considered a suitable instrument in this study, because it measures both prosocial and difficult behaviours in children. The combination of questions might enhance acceptability of the assessment among parents, teachers and nurses and makes it suitable for use in general populations where the majority of children are healthy. Furthermore, SDQ is validated on children aged 5–15, in Sweden [19], and norm data are available for children 2–5 years of age [20].

The SDQ contains 25 items that are divided between five subscales: hyperactivity/ concentration problems, behavioural problems, peer relationship problems, emotional symptoms and prosocial behaviours [21]. The SDQ takes about 5–10 minutes to complete. The SDQs efficiency in detecting social and emotional behavioural problems among older children has been proven [19,21,22], and the validity of a version adapted for 3- to 4-year-olds has also been examined, with satisfactory results [23]. The specificity of multi informant SDQs was 94.6% and the sensitivity 63.3% in a study conducted on a community sample of British 5- to 15-year-olds [22]. The weighted mean correlation between parents and teachers in a review [24] was 0.26–0.47 for the five subscales, with the best correlation for hyperactivity and behaviour problems.

### Using diffusion of innovation theory to understand how nurses, preschool teachers and parents experience the information sharing

Even when evidence is available, changes in practice can often turn out to be too complex to implement. Various theories have been developed to increase understanding of how changes in practice may be achieved [25]. Diffusion of innovations [26] is a theory in which innovations are defined as practices perceived as new by practitioners, and diffusion is defined as the spread of new practices among practitioners. According to the diffusion of innovations theory, there are five qualities of an innovation that determine the level of its adoption: *complexity*, *compatibility*, *relative advantage*, *observability* and *trialability*. The theory can be applied to understanding how characteristics of the new practice itself may aid its approval [27] and to identify barriers and enablers when implementing changes in practice. Therefore, this study aims to provide in-depth understanding of the experiences of nurses, preschool teachers and parents concerning the new method of information sharing using the SDQ, as well as to describe possible barriers and/or enablers when implementing this information sharing method.

This study is part of a comprehensive evaluation of the information sharing and mainly covers the implementation aspects. The new method’s clinical value and accuracy are under evaluation in separate studies.

## Methods

Grounded theory was employed in order to develop a theoretical model to describe how causal conditions and current context of child healthcare and preschool affect the introduction of information sharing through SDQ from the nurses, preschool teachers and parents’ perspectives. The grounded theory approach was considered to be beneficial as study design of data collection and data analysis since it allows not only theme generation but also an explanation and conceptualisation of the phenomenon at hand from different stakeholder perspectives. A total of 34 semi-structured interviews were conducted between March 2014 and June 2014 (nurses *n = *10, preschool teachers *n = 13*, parents *n = 11*).

### Recruitment and description of participants

Purposive and snowball sampling were used to recruit participants who had worked either as a nurse or a teacher for at least two years and had acquired experience with this new method of information sharing within the trial period. The participants were recruited from both publicly and privately run services (both of which are publicly financed).

Nurses were briefly informed about the study at a regular meeting and were asked to sign a list if they were willing to be interviewed about the new procedure. Out of approximately 45 nurses informed, 11 reported interest in being interviewed. Preschool teachers were recruited by snowball sampling: we asked our contact persons to recommend a colleague. About half of the teachers that were recommended wanted to participate. The individuals who abstained stated that they lacked either time or experience in the information sharing method.

Five nurses at three CHCs located in different areas recruited parents. During a period of two weeks, the nurses asked visiting parents with children aged 3–5-years-old if they were willing to be interviewed regarding the information sharing method using the SDQ, irrespective of whether they had filled out the SDQ or not before the visit. The total number of approached parents is unknown, and most parents who choose to participate had completed the SDQ.

The participating teachers and nurses worked in areas with varying socio-economic conditions. However, the interviewed parents’ individual socio-economic status is not known. All of the teachers and nurses were females. One of the parents was male. Ages ranged from 25 to 63 among teachers and nurses, and from 23 to 43 among parents. All nurses and parents were born in Sweden, and two of the preschool teachers were foreigners.

### Ethics

The trial was approved by the Regional Ethical Review Board in Uppsala (Dnr 2012/437). All of the interview data were anonymised to protect the identity of the informants. Participants were provided with a written information sheet prior to the interviews and allowed time to consider whether or not to participate in the study. The participants were also provided all pertinent information about the study verbally and given opportunity to ask questions at the time of the interview. Thus, verbal informed consent was obtained from all participants prior to commencing the recording of interviews. The Swedish Law on Ethics in Human Subjects [28] allows informed consent to be either verbal or in writing.

### Data collection and analysis

The first author of the study conducted the interviews with preschool teachers and parents. Two masters students conducted the interviews with nurses. Prior to the interviews, three interview guides were developed. The recorded interviews lasted between 15–50 minutes and took place in a private room at the teachers and nurses’ place of work or at the child’s CHC. One parental interview took place in a public place, according to the participant’s preferences. Interviews were transcribed verbatim. The variations in the interview length were partly attributable to the informants’ experiences in the information sharing method.

The analysis was based on the constant comparative method according to grounded theory, as described by Strauss and Corbin [29]. The process continued until no apparent new information emerged from the data. The first step of the analysis was open coding, where the interviews were coded sentence by sentence (Table 1). The codes were continuously sorted and compared until categories with the same information emerged. These categories were systematically compared to codes and categories from new data. The categories were then sorted and modified into more complex categories. Throughout the data collection and open coding, memoing was conducted. The memos served as reminders of the researcher’s thoughts about the meaning of the codes and categories in relation to the information sharing.

**Table 1:** Example of the open coding procedure, sentence by sentence.

**INTERVIEWER:**	**CODES:**
Why did you choose to fill in the form together with your colleague?	
**PRESCHOOL TEACHER** *:	
Uh… Yeah, but then because we work in teams… We are never at the preschool the whole day, and then, of course, we view children and situations differently… so, of course, we experience… and then we can get a broader… broader picture of a child… so, of course, we think in many situations… that… that what you see is perhaps not at all what I have experienced; my experiences may be completely different… when I am with the child. And that may result in… We may get a more accurate picture of the child, so to speak, than if it 's just me, for it is clear that it will be subjective perceptions… it…it will.	Teamwork
	See child part of day
	Different views
	More complete picture
	One teacher’s experience may not be the truth
	Fair picture of the child
	Subjective perceptions

*Interview with preschool teacher no 2.

Open coding was followed by axial coding, a procedure that seeks to identify how the categories relate to each other [30]. In this process, the categories were sorted into the building blocks of the emerging theoretical model, as suggested by Strauss and Corbin. These ‘blocks’ were: ‘causal conditions’, ‘context’, ‘strategies’ and ‘consequences’. The categories describing what had led to the phenomenon (information sharing) were sorted into ‘Causal conditions’, and the categories describing the conditions that shaped the ‘strategies’ (the actions or interactions) of people adopting the central phenomenon were sorted into ‘context’. Categories describing the result from the strategies were sorted into ‘consequences’. The final step was selective coding, in which the “core categories” were formulated on the basis of all the categories, characterising the central ideas about information sharing (phenomenon) among teachers, nurses and parents, i.e. all stakeholders.

Consultations were held within the research team on many occasions to decide about the next steps of data collection and to ensure that the analysis followed the constant comparative steps of grounded theory. Each researcher performed his/her own selective coding and then discussed their results in order to get a consensus. The theoretical model is a synthesis of the different selective coding patterns of the three authors.

## Results

**Fig 1:**
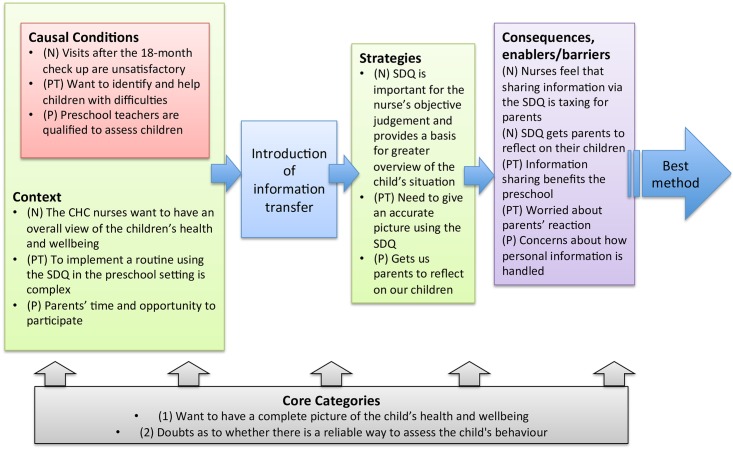
Theoretical model showing the interrelationships of the categories. Categories organised into the building blocks of the emerging theoretical model. Categories describing what had led to the information sharing were sorted into ‘Causal conditions’, and the categories describing the conditions that shaped the ‘strategies’ i.e. the actions or interactions of people adopting the central phenomenon were sorted into ‘context’. Categories describing the result from the strategies were sorted into ‘consequences’. The “core categories” were formulated on the basis of all categories, characterising the central ideas about information sharing among (N) Nurses, (PT) Preschool teachers and (P) Parents.

The three stakeholders’ experiences and thoughts about information sharing are shown in Table 2. Each subtitle represents a category label, and the labels are followed by whether the categories represent a causal condition, context, strategy or consequence in the axial coding. The final theoretical model showing the interrelationships of the categories is presented at the end of the results section (Fig 1). In the theoretical model, the relationship between the categories are visualised with arrows to show the direction in which the process proceeded; from the stakeholders’ need for information sharing to introduction of the process and subsequently towards a refined version of the information sharing process labelled ‘Best method’. The categories from respective stakeholders are described in more detail below, and selected quotes exemplify the content.

**Table 2:** Categories from the open and axial coding process for the three stakeholders.

Child Health Clinic-Nurses	Preschool teachers	Parents of 3–5-year-olds	Axial coding
Category label	Category label	Category label	
Visits after the 18-month check up are unsatisfactory	Want to identify and help children with difficulties	Preschool teachers are qualified to assess children	Causal condition
The CHC nurses want to have an overall view of the children’s health and wellbeing	To implement a routine using the SDQ in the preschool setting is complex	Parents’ time and opportunity to participate	Context
SDQ is important for the nurse’s objective judgement and provides a basis for greater overview of the child’s situation	Need to give an accurate picture using the SDQ	Gets us parents to reflect on our children	Strategy
Nurses feel that sharing information via the SDQ is taxing for parents	Information sharing benefits the preschool	Concerns about how personal information is handled	Consequence
SDQ gets parents to reflect on their children	Worried about parents’ reaction		

### Child health clinic nurses

#### Visits after the 18-month check up are unsatisfactory

The nurses argued that visits for 3- to 5-year-olds did not always reveal enough information about the child’s behaviour and that they lacked tools to identify even those children with obvious problems. The nurses also believed that the annual visits were short and not equal for all families since the content depends on whether the nurse knows the family, how much time is allocated for the visit, and what the parents choose to disclose and discuss. Therefore, nurses saw preschool teachers as a natural informant to involve in the behavioural assessment of preschool-aged children.

*‘We see the child for 30 or 45 minutes*, *and the preschool sees the child every day (*…*) Thus*, *they can make an assessment much better than we can’*

(Nurse no 6, public CHC).

The nurses felt it was important that the CHS was continuously updated about new scientific knowledge, due to the fact that the public has a lot of confidence in the CHS-system.

#### The CHS nurses want to have an overall view of the children’s health and wellbeing

The CHS nurses wanted to be a source of support for both children and parents and also to obtain an overall view of the children’s situation. The nurses argued that continuity was important for making accurate assessments.

#### SDQ is important for the nurse’s objective judgement and provides a basis for greater overview of the child’s situation

The nurses acknowledged SDQ as an important objective tool for their assessment and stated that it provided a basis for better overview of the child’s health and wellbeing. The nurses found that by using the SDQ, their discussions with parents became more structured and that valuable information was obtained.

*‘As I've gotten back the questionnaires and looked at them*, *I have discovered that you actually get more information (*…*) than you would have from a normal three-*, *four*, *or five-year-old check up’*

(Nurse no 3, private CHC).

Nurses found it very useful to be provided with completed SDQs from both parents and preschool teachers when assessing the child as the combination of parent and teacher SDQs made it easier to determine if the child was in need of further evaluation. They argued that parents and preschool teachers saw the child in different environments that entailed varying demands on the child. Several nurses also noted that important information about the child could be overlooked without the preschool assessments because parents were not always aware of their child's difficulties.

Nurses reported that parents’ and teachers’ responses tended to be consistent and also in line with the nurse’s own assessment. The questionnaire, thus, often functioned as a confirmation. The nurses found it valuable to see the preschool’s answers before the child’s visit because it made it possible for them to adjust the time required and to prepare relevant questions pertaining to issues raised in the preschool assessment.

#### Nurses feel that sharing information via the SDQ is taxing for parents

Nurses thought that requiring parents to fill out and bring their own SDQ, as well as handing the form over to the preschool, placed high demands on parents, thus, resulting in them not responding to the questionnaires.

‘If you have another mother tongue, even if you know Swedish, or if one is not as highly educated, then I feel that you are not as inclined to fill out these questionnaires’

(Nurse no 3, private CHC).

The nurses expressed that those families that did not fill out the SDQ might be the most important families to reach.

#### SDQ gets parents to reflect on their children

The nurses described that parents generally were very interested in the preschool assessments and that they put great emphasis on it. Parents were also keen to see that the preschool responses were appropriately followed up on by the CHS. Many nurses had heard from parents that SDQ got them to reflect on their children’s behaviour and has led to fruitful discussions between parents.

The nurses considered the responses from the preschools to be important information for parents either by making parents aware of problems or by calming parents who unnecessarily suspected problems.

### Preschool teachers

#### Want to identify and help children with difficulties

Many teachers expressed that the preschool had a responsibility to detect and act on the behavioural problems identified. Most preschool teachers reported that SDQ could be a good tool to identify children with mental health problems because of its structured and specific nature. Teachers felt that information sharing could expedite referrals and that families could get better support through cooperation between the preschool and the CHS. The teachers also suggested that assessments made by different parties were necessary when a complete picture was desirable. They also indicated the limits of their professional role and the need for better collaboration with the CHS.

*‘In the preschool*, *we are the professionals on children in groups and individual children*… *But children who have problems*, *who need support and such*, *we are not professionals in that*. *We know that there are problems*, *and we see signals*, *but then we need help*…*’*

(Preschool teacher no 7, Private preschool).

Nonetheless, some teachers believed that children with needs would be discovered in due course and that the questionnaires would not be of any help.

#### To implement a routine using the SDQ in the preschool setting is complex

All preschool teachers acknowledged that implementing SDQ in preschool is complex. They explained that teachers were ‘not allowed’ to make assessments with structured forms, according to the plan for preschool education and that the use of assessment forms were contradictory to the preschool’s philosophy. Many teachers felt uncertain as to whether to do the assessment at all, when the preschool director approved this procedure despite the perceived contradiction with the school policy documents.

*‘We have reacted a bit to that when it comes to filling out the questionnaire*, *because we shall not judge the children*. *We have agreed that we shall just see children as competent*. *And then it is like one fills out things that we really should not be doing then*, *we think*… *It's a bit against how we shall view the children’*

(Preschool teacher no 4, Public Preschool).

All teachers felt that their time was limited, but some expressed that the time to fill out the questionnaires could be well invested and manageable if they prioritised. Others argued that assessments should only be done when there was a specific need.

#### Need to give an accurate picture using the SDQ

The teachers regarded themselves as having competence to assess children’s behaviour. Furthermore, they claimed to know the children very well and believed that their experiences made them competent to notice deviant behaviours.

Many teachers considered SDQ to be adequate to provide a fair picture of the child. However, several teachers described fear of making incorrect judgements and had concerns about labelling the child. Some teachers thought that getting a picture of the child through structured forms could not be truly representative. To avoid bias, many teachers found it valuable to do the assessment together with colleagues, allowing them the opportunity to discuss different situations in which the child might have been observed.

*‘One can be afraid to put a negative label on a child when it really might all be about*… *uh*… *it could be just about anything*… *because what we see at the preschool is such a small part of the child's reality’*

(Preschool teacher no 1, Private preschool).

#### Information sharing benefits the preschool

Preschool teachers thought that filling out the SDQ could contribute by giving the preschool a more detailed picture of the child since the questions required careful reflections and generated valuable discussions between colleagues that could lead to supportive actions.

*‘It was good (*…*)*. *One had to really think about it and it was even more positive*, *since we did it together in the team*. *Because then*, *one can always get another picture too*. *Get the opportunity to discuss’*

(Preschool teacher no 13, Private preschool).

Some of the teachers thought that structured assessments could be good basis for discussion with the parents, especially in cases when the child had difficulties.

#### Worried about parents’ reactions

Teachers felt that parents were generally interested in the child’s social behaviour, but that it was not always easy to address parents’ reactions when discussing the child’s behaviour, either spontaneously when meeting the parents at the preschool or if parents had questions regarding the SDQ assessment at the developmental talk. Several teachers expected that assessments could lead to unpleasant conversations with parents and thought that some responses in the SDQ could make parents unnecessarily worried. They felt it was important that parents were informed about the purpose of the assessment and pointed out that obtaining parental consent was crucial, since the parents’ confidence in the preschool was central to their work. The vast majority of teachers had not experienced any negative reactions from parents since the onset of the information sharing method using the SDQ. However, some parents became worried when confronting the preschool’s assessment at the CHC and scheduled meetings with the teachers thereafter.

### Parents of 3–5-year-olds

#### Preschool teachers are qualified to assess children

The parents found the preschool teachers to be well educated and qualified to assess children’s behaviour. They believed that preschool evaluations could provide a complement to the parents’ description of the child and that opportunities to identify children in need of support increased through information sharing.

*‘Then I also think that it's important that*… *that the preschool also answers the same*. *For one can have a different picture as a parent and as a teacher*, *and then you can compare’*

(Parent interview no 9, Mother).

The parents did not see any problems with the preschool evaluations being sent to the CHC-nurse. On the contrary, they looked forward to finding out about the preschool teacher’s experiences and perceptions of their child. However, some of the parents acknowledged that they felt somewhat concerned that teachers would have different perception of their child’s behaviour than their own.

#### Parents’ time and opportunity to participate

Many parents considered the questions interesting and relevant. However, some parents expressed that answering the questionnaire was time-consuming and that certain questions were difficult to interpret. They saw these as possible reasons why some parents would choose not to respond. Informants felt there might be selection of parents who completed the forms, whereas large group of parents, who had language barriers or did not understand why they should fill out the questionnaire, would miss out.

#### Gets us parents to reflect on our children

Parents noted that the questions could get parents to reflect on their child and even see things they would not have identified otherwise. Many parents also noted that SDQ led to valuable discussions with the other parent. Some parents felt that the assessment only provided a snapshot of the child and that it was easy to give inaccurate answers unintentionally.

*‘It felt good*. *I thought that*… *it was a bit exciting to sit and fill it out (*…*)*. *Yet*, *there were some questions that I thought were formulated in a way so that they were difficult to answer’*

(Parent interview no 3, Mother).

The parents thought that their own responses and the preschools’ responses could be different, as the child’s behaviour varied depending on the environment and time of day. Some parents also described that their partner answered the questions differently than they themselves. They were of the opinion that both parents should answer the questions, as parents could interpret the questions differently and their relationship to the child may vary during different periods.

#### Concerns about how personal information is handled

Parents had concerns about how personal information was handled and how the answers would be used.

‘Of course, sometimes it was very private and it felt… So, yes, you kind of felt, what is this going to be used for?’

(Parent interview no 2, Father).

They expressed concerns about stigmatising their child and felt that having free text responses from the preschool might reduce the risk of minor problems being magnified. Accordingly, they wanted the preschool evaluations to be reviewed thoroughly by the nurse and professionally conveyed to the parents. Parents also expressed that they preferred to have their annual review discussion at the preschool, close to the CHC visit. This would give them opportunity to discuss the SDQ with the preschool teachers and thereby minimise risk of possible misunderstandings.

### Results from axial coding

During axial coding, the categories above were sorted into the building blocks of the emerging theoretical model: ‘causal conditions’, ‘context’, ‘strategies’ and ‘consequences’ related to information sharing.

In the final model, Fig 1, the interrelationships of the categories are visualised.

## Discussion

Overall, it seems that nurses, preschool teachers and parents share a mutual desire to (1) have complete picture of the child’s health and wellbeing, although they (2) have doubts as to whether there is a reliable way to assess children’s behaviour. Although information sharing through SDQ works in practise and causal conditions endorse introduction of information sharing regarding children’s mental health, successful implementation requires considerable work with regard to identified barriers.

### Applying diffusion of Innovations theory to understand barriers and enablers

The diffusion of innovations theory is a concept emphasising that the key to successful implementation is primarily about tailoring the innovation to fit the needs of different stakeholders. Our results reveal that nurses, preschool teachers and parents are, indeed, stakeholders with different needs and perceptions of the advantages of the innovation–information sharing through SDQ. According to the diffusion of innovations theory, there are five qualities of the innovation that determine the level of its adoption: *complexity*, *compatibility*, *relative advantage*, *observability* and *trialability*. These qualities are discussed below, in relation to our results.

Our findings indicate that structured assessments of children’s development are controversial within the preschool organisation—an aspect of *complexity* [27]. Even before initiation of the trial, preschool teachers expressed concern regarding them filling out the SDQ, as one of their policy documents [31] states that developmental screening methods based on normative principles of developmental psychology are not consistent with the preschool curriculum and pedagogical work [32]. The perception that developmental psychology-informed thinking about children is inadequate was also clearly prevalent among our informants, and teachers worried that normative thinking when using a scale would ‘label’ children as deviant with a potential negative impact on the child’s future. A qualitative study from Scotland found similar results where, although SDQ was found feasible as part of the routine transition process upon school entry, teachers raised concerns about the potential of labelling the child [14]. Thus, it would seem that structured assessment, as such, raises concerns for preschool teachers, across educational systems and contexts, about labelling. In fact, fear of labelling was the very word used in an evaluation of the Before School Check in New Zealand, where some church-related preschools rejected filling out the SDQ for their children [15], despite the structured assessment being rolled out as a routine in the whole country. Thus, it seems that implementation of a structured assessment instrument is considerably more *complex* in the educational setting than in the child healthcare setting. A possible explanation might be that nurses are used to working with different structured assessments and perceive them as a natural and essential part of their work and are therefore more willing to accept such tools.

Parents’ concern about how personal information was handled is another issue of *complexity*. Parents’ hesitation to provide data about their children is not surprising considering the public debate which indicates that many Swedish citizens feel uneasy about authorities’ potential use of personal information [33]. It must therefore be made clear to parents during the continued information sharing process that the data handling fully complies with ethical and legal rules.

The diffusion of innovations theory includes consideration of the innovation’s *compatibility* with existing values and practises. Innovations that are not compatible with the practitioners’ values and past experiences are less likely to be adopted [27]. Thus, concerns about labelling children and about parents’ reactions should be addressed. The Education Act is, in fact, quite explicit regarding the preschool’s mission to give children extra support for development, if needed [34]. Furthermore, the curriculum emphasises a well functioning collaboration between parents and preschool teachers as pivotal [35]. The findings in this study imply that national policy documents clarifying the value as well as the pitfalls of structured assessments can be critical to support implementing and maintaining information sharing through SDQ between educational and health institutions. This is going to be challenging: the existence of a definable “truth” through proper measurement is inherent in the biomedical paradigm, whereas the prevailing philosophy in preschool education in Sweden is closer to a constructivist approach, where the normative implications of standardised questionnaires are viewed as controversial or even undesirable—not *compatible* with current ideologies.

The diffusion of innovations theory also takes into account the innovation’s *relative advantage*. The nurses realised the benefits of information sharing; therefore, the rate of adoption among nurses may be more promising than among preschool teachers who do not perceive them as obvious benefits. Parents felt that different sources of assessment could complement each other, and they trusted and were interested in the preschool’s assessment of their child. These findings are in line with earlier research emphasising the importance of multiple informants when assessing child behaviour [22,24]. Furthermore, the results obtained in this study give reason to believe that parents’ recognition of the *relative advantage* can be enhanced given that the preschool SDQs are reviewed thoroughly by the nurse and professionally conveyed to them at the CHC visit. According to the findings in this study, parents might also perceive the *relative advantage* of the SDQ assessments to be considerable if they are able to have their annual review discussion at preschool close in time to the CHC visit, which would allow opportunity to ask questions regarding the preschool’s SDQ assessment. Overall, this study indicates that there seems to be a *relative advantage* of doing the assessment with, rather than without the SDQ. This is in accordance with previous studies, showing the successful use of teacher assessments in preschool using SDQs in other countries [36–38]. However, the *relative advantage* of using SDQ is not apparent regarding equity: not all parents completed the parent SDQs and even fewer brought the SDQ to the preschool. Nurses experienced that children whom they suspected as having problems and families with low socio-economic status often chose to abstain from completing the SDQs. These observations are troublesome since the association between socio-economic status and a number of different developmental and health outcomes is well established [10]. Inequity is thus a risk that should be taken seriously if proceeding with the information sharing method using the SDQ.

A further element of an innovation is *observability*. Preschool teachers could indeed *observe* that the SDQ could improve the quality of the developmental talks they held with parents; they felt that structuring their observations in the SDQ helped them to approach sensitive topics more easily. Nurses could also *observe* benefits as they experienced that the SDQ was important for their objective judgement and provided a useful tool in their conversations with parents. However, although the benefits were *observable* for preschool teachers and nurses, the efforts and deficits related to the new routine, such as inequity issues, could be considered as outweighing the advantages. Subsequently, the question might arise as to whether it is ethical and efficient to continue information sharing without any amendments. Extended marketing of the information sharing method might lead to greater awareness among parents regarding the benefits of the SDQ assessments and thus increase parents’ willingness to participate in the procedure. A well thought out marketing strategy could also have major impact, in terms of dealing with privacy issues among parents, if they learn from the marketing message that the information they share about their child will be handled according to all ethical regulations. Furthermore, it is important that parents understand that the SDQ assessments will not constitute basis for any diagnosis but serve only as a discussion document at the CHC visit.

In the diffusion of innovations theory, *trialability* is defined as the degree to which the innovation can be tested and modified to fit the users [26]. The information sharing method using the SDQ should, therefore, be evaluated and modified to fit parents’ expectations and become more user-friendly. An approach where the preschool teachers provide the CHC nurses with their SDQ assessments without the parents first bringing the questionnaires to the preschool could potentially make the procedure easier for parents. Such approach would require that the preschool obtain parental consent before sending the SDQ to the CHC but could possibly be a quite well functioning routine. Such modifications may increase participation among the particular group of families, who according to the nurses often chose to abstain from information sharing.

The introduction of the information sharing process has faced various challenges, and results from this study have led to a number of different efforts including re-designing the questionnaires, sending out newsletters to CHCs and having regular meetings with researchers and representatives from the municipality and County council. These meetings provide excellent opportunity to discuss known and newly identified problems regarding the information sharing process, and agree on solutions. Moreover, the meetings constitute an important information conduit since the representatives from the municipality and County council are able to forward information through the local managers who in turn forward the information to the preschool teachers/CHC-nurses. One of the main objectives with the meetings is to continue the dialogue regarding how to manage teachers' resistance to completing standardised forms. The findings obtained in this study can be used to bring about change in that matter if preschools, local managers and teachers are informed about CHC-nurses’ perception that the preschool SDQ’s are important for their assessment, and that parents found the preschool teachers to be qualified to assess children. Furthermore, the teachers’ resistance could also be reduced if all preschool teachers are informed about the specific strategy already used by some preschool teachers: to avoid bias by doing the assessment together with colleagues. This strategy is a suitable way to fulfil preschool teachers’ *need to give an accurate picture using the SDQ* (Fig 1), which could be beneficial for most preschool teachers. Moreover, in order to make preschool teachers feel more comfortable with the SDQ assessment it might be crucial to, just like with parents, clarify to all preschool teachers that their SDQ reports will only serve as basis of discussion at the CHC and not be used for diagnostic purposes. The teachers’ concerns about parents’ reactions should also be addressed. One way to address this could be to minimise the risk of parents becoming worried or upset when confronting the preschool’s assessment at the CHC, which is also necessary from a parental perspective. That is, the preschool SDQs should, as far as possible, not contain any information that has not already been disclosed to the parents by the preschool teachers themselves. The possibility of parents getting “news” regarding their child’s behaviour through the preschool SDQ was considered when designing the information sharing process. However, since the preschool leadership argued that preschool teachers always raise any problems regarding the child with the parents as soon as the problems are identified, the risk of this problem might have been underestimated. In fact, findings in this study indicate that this specific problem has occurred since the introduction of the information sharing process. Thus, preschool teachers need to be reminded of the importance of them raising any issues with the parents before sending the completed SDQ to the CHC. In conclusion, a well functioning information conduit to provide preschool teachers with information and reminders is most likely of great importance for the long-term maintenance and success of the information sharing process.

In this study, information sharing was performed using the Strengths and Difficulties Questionnaire (SDQ) [17], which had been assessed by parents and preschool teachers. However, it is possible that the findings would be similar if another structured assessment tool for children’s development had been used. Perhaps, the barriers identified relate more to the use of the structured assessments of children’s development as a phenomenon and less about the specific instrument itself.

### Methodological considerations

#### Transferability

Participants represented a variety of ages, ethnicities and socio-economic backgrounds. Furthermore, saturation was reached for all stakeholders. The findings may, therefore, be transferable to the broader Swedish setting. However, we know that only 50% of parents in the target population participated in the information sharing process using SDQ; thus, more knowledge is needed about the perceptions of parents who opt out. Also, not all approached nurses and preschool teachers agreed to participate. Most of them lacked experience with the SDQ, but some cited lack of time as a reason. However, it is possible that preschool teachers and nurses who agreed to participate were more positive towards information sharing; thus, a possible selection bias cannot be ruled out.

#### Credibility

To minimise the effect of the interviewer being a nurse or nursing student, an interview guide was used, three analysts participated in the analysis, and results were discussed with representative stakeholders. The theoretical model was discussed in detail among peers.

#### Dependability

Grounded theory focuses on generating theoretical ideas exclusively from the data [39]. It is our understanding that the theory developed in this study is the product of a process, carefully following the principles of grounded theory through Strauss and Corbin’s (1998) approach.

## Conclusions

The theoretical model developed describes that the causal conditions and current context of child healthcare in many respects endorse the introduction of information sharing through SDQ from the nurses’, preschool teachers’ and parents’ perspectives. However, a successful and sustainable implementation requires considerable work in addressing the identified barriers, including the tensions between normative thinking versus helping children with developmental problems for preschool teachers and dealing with privacy issues and inequity in parents’ participation. In addition, efforts to reach more vulnerable groups in society need to be a priority. Extended marketing of the information sharing method and modifications of the process in order to make it less taxing for parents might lead to increased willingness and confidence among parents in all socioeconomic groups to participate.

## Supporting Information

S1 File Codebook. (DOC)

S2 File Survey Questions in English. (DOCX)

S3 File Survey Questions in Swedish. (DOCX)
