# Supplementary material for: Exploring Nurses’, Preschool Teachers’ and Parents’ Perspectives on Information Sharing Using SDQ in a Swedish Setting – A Qualitative Study Using Grounded Theory
Source: PLoS One. 2017 Jan 11;12(1):e0168388. doi: 10.1371/journal.pone.0168388 (PMC5226714; doi:10.1371/journal.pone.0168388)
Supplement: S1 File — (DOC) [file pone.0168388.s001.doc]

**Codebook.**

| **Stakeholder** | **Major categories** | **Sub categories** | **Definition** |
| --- | --- | --- | --- |
| **Child Health Clinic-Nurses** | Visits after the 18-month check up are unsatisfactory | The CHC visits need to be updated | CHC nurses discuss various types of shortcomings of the visits for 3- to 5-year-olds. |
|  |  | The CHC visits are not equal for all families | Participants report that visits were short and not equal for all families since the content depends on multiple factors. |
|  | The CHC nurses want to have an overall view of the children’s health and wellbeing | Support for both children and parents | Participants discuss their aim to be a source of support for both children and parents and also to obtain an overall view of the children’s situation. |
|  |  | Continuity | CHC nurses report on how continuity is important for making accurate assessments. |
|  | SDQ is important for the nurse’s objective judgement and provides a basis for greater overview of the child’s situation | SDQ provides valuable information | CHC nurses acknowledge SDQ as an important objective tool for their assessment. |
|  |  | SDQ provides greater overview | CHC nurses experience that SDQ provides a basis for better overview of the child’s health and wellbeing. |
|  |  | ‘Information received from both parents and teachers’ | Nurses found it very useful with information from both parents and preschool teachers. |
|  |  | Parents and teachers’ perceptions of the child | CHC nurses discuss how parents and preschool teachers observe the child in different environments that place varying demands on the child. |
|  |  | Important information could be overlooked | Important information about the child can be overlooked without the preschool assessments because parents are not always aware of their child's difficulties. |
|  |  | Easier to raise sensitive issues | By using the SDQ, discussions with parents become more structured, and valuable information can be obtained since the structured form makes it easier to raise sensitive issues. |
|  | Nurses feel that sharing information via the SDQ is taxing for parents | The preschool questionnaire is a complicated procedure | Participants discuss how the complicated procedure for the SDQs (from the parents to the preschool to the CHC) can result in parents not responding to the questionnaires. |
|  |  | Not all parents participate | CHC nurses report that certain families do not fill out the SDQ. |
|  |  | Selection by the nurse | Sometimes CHC nurses choose not to send the SDQ questionnaires to families they find particularly vulnerable or not as inclined to fill out the questionnaires. |
|  | SDQ gets parents to reflect on their children | Parent’s are not always aware | The responses from the preschools can make parents aware of problems. |
|  |  | Gets parents to reflect | CHC nurses hear from parents that SDQ got them to reflect on their children’s behaviour and has led to fruitful discussions between parents. |
|  |  | The preschool assessments is important for parents | Parents are generally very interested in the preschool assessments and they put great emphasis on it. |
| **Preschool teachers** | Want to identify and help children with difficulties | Identify children with difficulties | Participants suggest that SDQ might be a good tool to identify children with mental health problems because of its structured and specific nature. |
|  |  | Obligation to detect and act | Preschool teachers express the preschool’s responsibility to detect and act on the behavioural problems identified. |
|  |  | Assessments made by different parties | Teachers suggest that assessments made by different parties are necessary when a complete picture is desirable. |
|  |  | Collaboration with the CHS | Participants indicate the limits of their professional role and the need for better collaboration with the CHS. |
|  | To implement a routine using the SDQ in the preschool setting is complex | Contradictory to the preschool’s philosophy | Teachers perceive structured assessment forms to be contradictory to the preschool’s philosophy. |
|  |  | Assessments in preschool | Participants discuss how, according to the preschool philosophy, they shall not judge the children, but see them as competent individuals. |
|  |  | Time constraints | Teachers’ perceptions regarding time constraints and the new routine with SDQ assessments. |
|  |  | The decision to perform SDQ assessments | Teachers feel uncertain as to whether to do the SDQ assessment, when the preschool director approved this procedure despite the perceived contradiction with the school policy documents. |
|  | Need to give an accurate picture using the SDQ | A fair picture using the SDQ | Teachers discuss whether the SDQ is adequate to provide a fair picture of the child. |
|  |  | Concerns about labelling the child | Teachers describe a fear of making incorrect judgements and had concerns about labelling the child. |
|  | Information sharing benefits the preschool | A more detailed picture of the child | Teachers report that filling out the SDQ might contribute by giving the preschool a more detailed picture of the child since the questions required careful reflections. |
|  |  | A fair picture of the child | Teachers report that filling out the SDQ together in the team might contribute to a fair assessment of the child because then, the teachers get the opportunity to discuss. |
|  |  | Valuable discussions | Teachers report that the SDQ routine generates valuable discussions between colleagues that might lead to supportive actions. |
|  |  | A good basis for discussion with the parents | Teachers discuss how the SDQ assessment could be a good basis for discussion with the parents, especially in cases when the child has difficulties. |
|  | Worried about parents’ reaction | Parental consent is crucial | Participants’ perceptions regarding the importance of obtaining parental consent. |
|  |  | Expects resistance and concerns among parents | Participants’ thoughts regarding how to address parents’ reactions when discussing the child and the SDQ assessment. |
|  |  | Experiences from parents’ reactions | Teachers’ experiences of reactions from parents regarding the SDQ assessment. |
| **Parents of 3–5-year-olds** | Preschool teachers are qualified to assess children | Qualified to assess children | Parents find the preschool teachers to be well educated and qualified to assess children’s behaviour. |
|  |  | The discussions with parents at the CHC | Participants’ thoughts regarding finding out about the preschool teacher’s experiences and perceptions of their child at the CHC visit. |
|  | Parents’ time and opportunity to participate | The information sharing requirements | Parents report that answering the questionnaire is time-consuming and that certain questions might be difficult to interpret. |
|  |  | The SDQ questions | Parents comment on the SDQ questions. |
|  | Gets us parents to reflect on our children | An eye-opener | The SDQ can get parents to reflect on their child and even see things they would not have identified otherwise. |
|  |  | Valuable discussions | Parents note that SDQ led to valuable discussions with the other parent. |
|  | Concerns about how personal information is handled | Stigmatising my child | Parents express concerns about stigmatising their child. |
|  |  | Risk of mis-understandings | Parents discuss possibilities to reduce the risk of possible misunderstandings regarding the preschool evaluation. |
|  |  | How to convey the preschool evaluation | Parents want the preschool evaluations to be reviewed thoroughly by the nurse and professionally conveyed to the parents. |
