# Supplementary material for: Exploring Nurses’, Preschool Teachers’ and Parents’ Perspectives on Information Sharing Using SDQ in a Swedish Setting – A Qualitative Study Using Grounded Theory
Source: PLoS One. 2017 Jan 11;12(1):e0168388. doi: 10.1371/journal.pone.0168388 (PMC5226714; doi:10.1371/journal.pone.0168388)
Supplement: S2 File — (DOCX) [file pone.0168388.s002.docx]

**Interview guide for nurses**

**Purpose:** To investigate nurses' experience of working with the SDQs.

Initially, there is a repetition of the purpose of the study, confidentiality, the right to withdraw, etc. Thereafter, consent is obtained for audiotaping.

1. How long have you used the questionnaires?
2. How much experience do you have working as a nurse at the Child Health Clinic?
3. How do you view your role at the 3-, 4- and 5-years visits?
4. How is it to use the SDQs? What is positive? What is negative?
5. Do you feel that the questionnaire has influenced / changed your assessment of any child? If yes, can you give an example?
6. Can you tell us about a time when you were really satisfied with the visit according to the new routine? Why was that?
7. Can you tell us about a time when it has not worked so well with the visit according to the new routine? Why was that?
8. Have you gotten any additional information about a child through the SDQs?
9. Has the new procedure affected your way of work in any way? If yes, for the better / worse?
10. What do you do if the parent’s and the preschool’s answers are not consistent? Have you received any directive on how to act in that situation?
11. How do you perceive the parents' reactions on the questionnaire?
12. How is it to have access to the pre-school assessment before you meet the child? Does it influence the visit in any way?
13. Do you feel that your assessment of the child is affected by having access to the preschool’s answers about the child?
14. What do you do if the last question (Question 26)^^[[1]](#footnote-1)^^ is answered with "Yes, severe difficulties"?
15. Is it possible to integrate the new routine (using SDQ) in your daily work?
16. Any other thoughts / experiences?

**Interview guide for preschool teachers**

**Purpose:** To investigate preschool teachers' experience of working with the SDQs.

Initially, there is a repetition of the purpose of the study, confidentiality, the right to withdraw, etc. Thereafter, consent is obtained for audiotaping.

1. How is it to use the questionnaire?
2. Do you think that you were able to provide the nurse at the Child Health Clinic a fair picture of the child by filling in the questionnaire?
3. Has the questionnaire changed your assessment of any child? If yes - Could you

give an example?

1. What’s it like to fill out the SDQ?
2. Do you think that is takes a lot of time to fill out the SDQs?
3. Has the new routine affected your work with the children in any way?
4. How do you perceive the parents' reaction to the questionnaire?
5. Do you think that most parents will participate in the study? Why? / Why not?
6. Do you think your assessment usually corresponds with the parents' assessment?
7. In cases where you have signalled that the child's behaviour / development is different from the other preschool children’s:

- How does it feel to know that the information will be received by the nurse at the Child Health Clinic? Why?
- Do you think your work with the child at the preschool can be facilitated later on as a result of the fact that the nurse will see your assessment of the child?

1. Do you think that assessments using structured questionnaires should be used in the preschool? Please tell us what you think about this.
2. How do your colleagues perceive the new working routine?
3. Any other thoughts / experiences?

**Interview guide for parents**

**Questions for parents who have decided to fill out the questionnaire**

**Purpose:** To investigate parents' experience of filling out the SDQ.

Initially, there is a repetition of the purpose of the study, confidentiality, the right to withdraw, etc. Thereafter, consent is obtained for audiotaping.

1. How old is your child referred to in the questionnaire?
2. Please tell us a little about your family. Do you live together with the child's father/mother or another adult? Does your child have siblings? Is the family’s mother tongue Swedish? Is the child born in Sweden?
3. What was your initial reaction when you got the letter with the questionnaire?
4. Do you think the purpose of the study was clearly described in the study information?
5. Was it difficult to understand how you should fill out the questionnaire?
6. Was it difficult to understand who should fill out which of the questionnaires?
7. How did it feel to fill out the questionnaire?
8. What did you think about the questions? Was there any specific question that you found difficult to answer?
9. Has the questionnaire changed your assessment of your child?
10. How do you feel about the fact that the preschool teachers will complete a questionnaire about your child and that the answers will be sent directly to the Child Health Clinic?
11. Did you discuss the questionnaire at the Child Health Clinic? If yes, how did you feel about the discussion?
12. How do you feel about the preschool teachers’ reactions to the questionnaire?
13. Do you think that preschool teachers should assess children with this type of questionnaire?
14. Have you talked to / heard from other parents about the questionnaire? If yes, how do you think they experience this procedure?

**Interview guide for parents**

**Questions to parents who chose not to participate:**

**Purpose**: To investigate parents' experience of filling out the SDQ.

Initially, there is a repetition of the purpose of the study, confidentiality, the right to withdraw, etc. Thereafter, consent is obtained for audiotaping.

1. How old is your child referred to in the questionnaire?
2. Could you tell us a little about your family. Do you live together with the child's father/mother or another adult? Does your child have siblings? Is the family’s mother tongue Swedish? Is the child born in Sweden?
3. What was your spontaneous reaction when you got the letter with the questionnaires?
4. What was the reason that you chose not to participate?
5. Do you think the purpose of the study was clearly described in the study information?
6. You have chosen not to participate in the study, but did you fill out the questionnaire anyway? (To provide the nurse with the SDQ information without participating in the study.)

- Why did you do that? / Why did you not do that?

**If the questionnaire was filled out:**

- Did you leave the questionnaire with the preschool, or did you just fill out the parent’s questionnaires?

(If relevant: Why didn’t you leave the questionnaire with the preschool?)

(If relevant: How do you feel about the preschool’s reactions to the questionnaire?)

- Do you think that preschool teachers should assess children with this type of questionnaire?
- Was it difficult to understand how you should fill out the questionnaire?
- Was it difficult to understand who should fill out which of the questionnaires?
- How did you feel about filling out the questionnaire?
- How did you feel about the questions? Was there any specific question that you found odd or difficult to answer?
- How do you feel about the fact that the preschool teachers will complete a questionnaire about your child and that the answers will be sent directly to the Child Health Clinic?
- Did you discuss the questionnaire at the Child Health Clinic? If yes, how did you feel about the discussion?
- Has the questionnaire changed your assessment of your child?

1. Have you talked to / heard from other parents about the questionnaire? If yes, how do you perceive their experience of the procedure with the questionnaire?
2. What do you think would get more parents to participate in the study?

1. The question is: Overall, do you think that your child has difficulties in one or more of the following areas: emotions, concentration, behaviour or being able to get on with other people? [↑](#footnote-ref-1)
