# Supplementary material for: Exploring Nurses’, Preschool Teachers’ and Parents’ Perspectives on Information Sharing Using SDQ in a Swedish Setting – A Qualitative Study Using Grounded Theory
Source: PLoS One. 2017 Jan 11;12(1):e0168388. doi: 10.1371/journal.pone.0168388 (PMC5226714; doi:10.1371/journal.pone.0168388)
Supplement: S3 File — (DOCX) [file pone.0168388.s003.docx]

**Intervjuguide för sjuksköterskor**

**Syfte:** Att undersöka sjuksköterskors upplevelse av att arbeta med SDQ.

Inledningsvis repetition av syfte, konfidentialitet, rätt att avbryta mm. Därefter inhämtas samtycke till bandupptagning.

1. Hur länge har ni använt er av formulären?
2. Hur lång erfarenhet har du av att arbeta som sjuksköterska på barnavårdscentralen (BVC)?
3. Hur ser du på din roll vid 3-, 4- och 5-års besök?
4. Hur är det att använda sig av formulären? Vad är positivt? Vad är negativt?
5. Upplever du att formuläret påverkat/förändrat din bedömning av något barn? Om ja, kan du ge ett exempel?
6. Kan du berätta om ett tillfälle när du varit riktigt nöjd med besöket enligt den nya rutinen? Varför var det så?
7. Kan du berätta om ett tillfälle när det har fungerat mindre bra med besöket enligt den nya rutinen? Varför var det så?
8. Har du fått extra information om något barn genom frågeformulären?
9. Har den nya proceduren påverkat ditt arbete med barnen på något sätt? Om ja, till det bättre/sämre?
10. Hur gör du om föräldrarnas och förskolans svar inte är överensstämmande? Har du fått några direktiv hur du ska agera om en sådan situation uppstår?
11. Hur upplever du föräldrarnas reaktioner på formuläret?
12. Hur är det att redan ha tillgång till förskolans bedömning innan du träffar barnet? Gör det någon skillnad vid besöket?
13. Upplever du att din bedömning av barnet påverkas av att få in förskolans svar om barnet?
14. Hur gör du om sista frågan (fråga 26)^^[[1]](#footnote-1)^^ besvaras med "- Ja, stora problem"?
15. Hur går det att integrera det nya arbetssättet (med SDQ) i ert dagliga arbete?
16. Övriga funderingar/erfarenheter?

**Intervjuguide för förskolepersonal**

**Syfte:** Att undersöka förskolepersonals erfarenheter av att arbeta med SDQ.

Inledningsvis repetition av syfte, konfidentialitet, rätt att avbryta mm. Därefter inhämtas samtycke till bandupptagning.

1. Hur tycker du att det går att fylla i formuläret?
2. Tycker du att du kunnat ge barnavårdscentralen (BVC) en tydlig och rättvis bild av barnet genom att fylla i formuläret?
3. Har formuläret förändrat din bedömning av något barn

Om ja – Kan du ge ett exempel?

1. Hur upplever du det är att fylla i SDQ?
2. Tycker du att det tar lång tid att fylla i formulären?
3. Har den nya proceduren påverkat arbetet med barnen på något sätt?
4. Hur upplever du föräldrarnas reaktion på formuläret?
5. Tror du att de flesta föräldrarna kommer att delta i studien? Varför? / Varför inte?
6. Tror du att din bedömning oftast stämmer överens med föräldrarnas bedömning?
7. I de fall då du signalerat att barnets beteende/utveckling är annorlunda än hos de andra barnen på förskolan:

- Hur känns det att veta att informationen kommer BVC-sköterskan till handa? Varför?
- Tror du att ert arbete med barnet på förskolan i förlängningen kan underlättas genom att BVC får se er bedömning av barnet?

1. Tycker du att bedömningar med strukturerade formulär hör hemma i förskolan? Berätta hur du tänker kring detta.
2. Hur upplever dina kollegor det nya arbetssättet?
3. Övriga funderingar/erfarenheter?

**Intervjuguide för föräldrar**

**Frågor till föräldrar som har valt att besvara formulären**

**Syfte:** Att undersöka föräldrars erfarenheter av att besvara SDQ.

Inledningsvis repetition av syfte, konfidentialitet, rätt att avbryta mm. Därefter inhämtas samtycke till bandupptagning.

1. Hur gammalt är ditt barn som formulären gäller?
2. Kan du berätta lite om din familj. Lever du tillsammans med barnets pappa/mamma eller annan vuxen person? Har ditt barn syskon? Har familjen svenska som modersmål? Är barnet fött i Sverige?
3. Vilken var din spontana reaktion då du fick hem brevet med formuläret?
4. Tycker du att studiens syfte framgick tydligt i studieinformationen?
5. Tycker du att det var svårt att förstå hur du skulle fylla i formuläret?
6. Tycker du att det var svårt att förstå vem som skulle fylla i vilket formulär?
7. Hur kändes det att fylla i formuläret?
8. Hur upplevde du frågorna? Var det någon fråga som du tyckte var svår att besvara?
9. Har formulären förändrat din bedömning av ditt barn?
10. Hur känner du inför att förskolan ska besvara ett formulär om ditt barn och att svaren sedan skickas direkt till barnavårdscentralen (BVC)?
11. Diskuterade ni formuläret på BVC? Om ja, Hur upplevde du diskussionen?
12. Hur upplever du förskolans reaktioner på formuläret?
13. Tycker du att förskollärare ska bedöma barn med den här typen av formulär?
14. Har du pratat med/hört något från andra föräldrar angående formuläret? Om ja, vilken tror du att deras upplevelse av proceduren är?

**Intervjuguide för föräldrar**

**Frågor till föräldrar som valt att inte delta:**

**Syfte:** Att undersöka föräldrars erfarenheter av att besvara SDQ.

Inledningsvis repetition av syfte, konfidentialitet, rätt att avbryta mm. Därefter inhämtas samtycke till bandupptagning.

1. Hur gammalt är ditt barn som formulären gäller?
2. Kan du berätta lite om din familj. Lever du tillsammans med barnets pappa eller annan vuxen person? Har ditt barn syskon? Har familjen svenska som modersmål? Är barnet fött i Sverige?
3. Vilken var din spontana reaktion då du fick hem brevet med formulären?
4. Vilken var anledningen till att du/ni valde att inte delta?
5. Tycker du att studiens syfte framgick tydligt i studieinformationen?
6. Du har valt att inte delta i studien, men har du fyllt i formuläret ändå? (För att ge informationen till sjuksköterskan på barnavårdscentralen utan att delta i studien.)

- Varför gjorde du det? / Varför gjorde du inte det?

**Om formuläret besvarats:**

- Lämnade du formuläret till förskolan, eller besvarade ni bara föräldraformulären?

(Om aktuellt: Varför lämnade du inte formuläret till förskolan?)

(Om aktuellt: Hur upplever du förskolans reaktioner på formulären?)

- Tycker du att förskollärare ska bedöma barn med den här typen av formulär?
- Var det svårt att förstå hur du skulle fylla i formuläret?
- Var det svårt att förstå vem som skulle fylla i vilket formulär?
- Hur kändes det att fylla i formuläret?
- Hur upplevde du frågorna? Fanns det någon fråga som du tyckte var märklig eller svår att besvara?
- Hur känner du inför att förskolan ska besvara ett formulär om ditt barn och att svaren sedan skickas direkt till barnavårdscentralen (BVC)?
- Diskuterade ni formuläret på BVC? Om ja, Hur upplevde du diskussionen?
- Har formulären förändrat din bedömning av ditt barn?

1. Har du pratat med/hört något från andra föräldrar angående formuläret? Om ja, Hur uppfattar du deras upplevelse av proceduren med formuläret?
2. Vad tror du skulle få fler föräldrar att delta i studien?

1. Frågan lyder: Tycker du att ditt barn har svårigheter inom något av följande områden: känslor, koncentration, beteende eller komma överens och umgås med andra människor? [↑](#footnote-ref-1)
